# Ecological Modules Link Soil Aggregate Stability, Chemical Properties and Fungal Communities Under Plant Species‐Based Revegetation

**DOI:** 10.1111/1758-2229.70228

**Published:** 2025-11-04

**Authors:** Zijian Ding, Jiahuan Li, Long Bai

**Affiliations:** ^1^ College of Horticulture, Shenyang Agricultural University Shenyang China

**Keywords:** fungal community, grassland species, microbial network, soil aggregate stability, soil chemical properties, trophic mode

## Abstract

The establishment of native grassland species is widely implemented on abandoned land as a strategy to restore degraded soils. However, its effects on soil properties are highly species‐specific, as plant‐driven physicochemical changes subsequently reshape microbial community structure. The linkages between soil physicochemical properties and microbial communities following native grassland establishment remain poorly understood. To address this knowledge gap, we examined the effects of 11 native grassland species on soil physicochemical properties and fungal community structure. Using co‐occurrence network analysis, we elucidate how plants drive fungal community reorganisation through soil‐mediated trophic pathways. The results showed that soil aggregate stability, chemical properties, and fungal communities differed significantly among the 11 species. Soil chemical properties, such as pH and EC, correlated with symbiotic fungi dominated modules; both soil aggregate stability and chemical properties were linked to pathogenic fungi dominated modules, while saprophytic fungi dominated modules displayed no linkage to either soil aggregate stability or chemical properties. These findings establish that fungal trophic modes govern species‐dependent restoration outcomes via modular soil–microbe linkages, thereby offering predictive frameworks for species‐specific management of abandoned soils.

## Graphical abstract

Under plant species‐based revegetation, network analysis revealed that fungal communities exhibited modular responses to variations in soil aggregates and chemical properties. Soil chemical properties shaped symbiotic fungi‐dominated modules; soil aggregates and chemical properties shaped pathogenic fungi‐dominated modules; while saprophytic fungi‐dominated modules were unaffected by either factor.

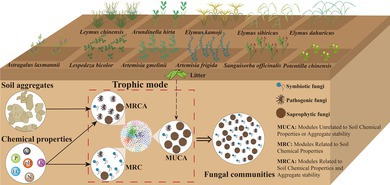

## 1 Introduction

Land degradation has emerged as a prominent global concern, receiving considerable attention. The combined effects of excessive exploitation and land use change, along with the impacts of global climate change, have significantly contributed to the degradation (Borrelli et al. 2020; Smith et al. 2016). Soil microbial communities are central to maintaining soil fertility and supporting crop production (Wang et al. 2018). Land degradation disrupts these communities, thereby reducing soil suitability for crop growth and making recovery difficult in the short term (Hartmann and Six 2023). The establishment of native grassland species is widely practiced on abandoned lands to alleviate degradation and represents an effective, economical approach to soil ecological rehabilitation (Chen et al. 2015; Deng et al. 2014). However, the effects of native species planting largely depend on the specific grassland species used, as their traits shape soil physicochemical properties and modulate soil microbial communities (Hobbie 2015; Jin et al. 2017; Zemunik et al. 2015). Consequently, there is a clear need for comprehensive studies that examine how plant species alter soil properties and microbial communities, and their functional interdependencies.

Soil fungi play important roles in soil degradation and recovery processes, particularly in the formation and stabilisation of soil aggregate stability under land use change (Zhu et al. 2022). Soil aggregates serve as a refuge for fungi, offering protection from predators and creating an adequate habitat for soil fungal growth (Erktan et al. 2020). The foraging behaviour of soil fungi and the spatial exploration of hyphae are also influenced by the pore structure of the soil (Aleklett et al. 2021; Harris et al. 2006). Likewise, soil chemical properties can influence the abundance of fungi by modifying the environmental conditions of the soil (Camenzind et al. 2018). For example, an increase in the soil available nutrients may result in the stimulation of fungal growth (Hammer et al. 2011). Generally, changes in soil aggregate stability and chemical properties tend to occur simultaneously and consistently (Iskandar et al. 2022; Ma, Hu, et al. 2022); these changes may mediate soil fungal communities collectively or individually. The alteration of fungal communities driven by soil aggregate stability and chemical properties is a complex process, the full understanding of which is still evolving.

Soil fungi encompass a variety of trophic modes, including pathotroph, saprotroph, and symbiotroph, which reflect the diverse ways fungi acquire energy and nutrients (Nilsson et al. 2019). These trophic modes respond differently to changes in soil physicochemical properties. For example, a deficiency of soil phosphorus can lead to a decrease in the abundance of symbiotic fungi, which gives rise to an increase in pathogenic fungi (Mujica et al. 2020). In recent years, a growing body of evidence has shown that the influence of soil physicochemical properties on fungal communities varies with the trophic modes (Chen et al. 2022; Yang et al. 2022; Zhang et al. 2021). As a result, changes in the trophic modes of fungal communities serve as a good indicator to explain the complex relationship between soil physicochemical properties and fungal communities.

Microorganisms do not exist in isolation but often form closely interacting ecological modules within the soil microbiome (Röttjers and Faust 2018; Toju et al. 2016). These modules may represent fundamental ecological units in terms of both function and diversity (Dundore‐Arias et al. 2023). Therefore, we hypothesize that these modules are more sensitive in terms of the response of soil physicochemical properties than the entire fungal community. Moreover, the interactions between microorganisms are closely related to the niche complementarity and overlap among the trophic modes of fungi (Albornoz et al. 2022). We further hypothesize that the response of the modules to soil physicochemical properties is related to the trophic mode of the fungi in these modules.

In this study, we cultivated 11 grassland species on abandoned cropland for two growing seasons to evaluate the influence of plant species on soil fungal communities, through the changes in soil aggregate stability and chemical properties respectively. Our objective is to investigate how the fungal communities respond to the changes in soil aggregate stability and chemical properties regulated by plant species, specifically through the perspective of network modules. Focusing on this objective, we investigated the following research questions: (1) Do soil fungal communities exhibit a response to changes in soil aggregate stability and chemical properties caused by plant species? (2) Do soil fungal communities exhibit modularity in relation to soil aggregate stability or chemical properties within a co‐occurrence network? (3) Do soil aggregate stability and chemical properties respectively or jointly regulate certain microbial modules? Are the relationships between them linked with fungal trophic modes?

## 2 Materials and Methods

### 2.1 Field Site

This study was carried out at Fuxin Mongolian Autonomous County, Fuxin, Liaoning, China (30^◦^ 53′ 41.9” N, 121^◦^23′ 15.8” E), a long‐term experimental site we set up with local forest and grass authorities. The climate is temperate semi‐arid continental monsoon with a mean precipitation of 478.9 mm and a temperature of 7.7°C. The soil at this site is classified as Dystric Cambisol according to the Food and Agriculture Organization of the United Nations classification. The mean annual evaporation is approximately 1848 mm, and the growth period lasts 173 days. Historically, the land under study was originally covered by grassland vegetation. About 20 years ago, the grasslands of this region were reclaimed on a large scale for maize fields. Over time, the effects of climate change, soil degradation and human activities have led to the emergence of large tracts of abandoned cropland. Our experiment was conducted at a large abandoned area that had been historically managed as monoculture maize farmland. During the annual spring sowing period, approximately 750 kg hm^−2^ of an N‐P_2_O_5_‐K_2_O compound fertiliser is applied to the cropland. After the final maize harvest and removal of crop residues the year before (2020), the field was left fallow.

### 2.2 Field Experiment Design and Soil Sampling

The 11 grassland species selected for our experiment are typical species in local grasslands or species used for local ecological restoration, including *Leymus chinensis, Arundinella hirta, Elymus kamoji, Elymus sibiricus, Elymus dahuricus, Astragalus laxmannii, Lespedeza bicolor, Artemisia gmelinii, Artemisia frigida, Sanguisorba officinalis, Potentilla chinensis*. Their seeds were collected locally. To ensure the successful establishment of young plants, we utilize a seedling hole tray (each pot: bottom diameter 2 cm, top diameter 3 cm, height 4 cm) in a greenhouse for seed germination. When the seedlings reach a height of 15–20 cm, they are transplanted to the field. A total of nine seedlings were transplanted per square meter, arranged in a 3 × 3 grid with approximately 30 cm spacing between plants in both directions. The transplanting was completed in June 2021, with each plant species being planted in a designated 100 m^2^ area (10 × 10 m). No fertiliser was applied.

After two growing seasons, 0–20 cm soil samples were collected on July 25, 2022. At this time, the plant cover generally reached over 80%, with an average plant height of approximately 100 cm, and the biomass (fresh grass) was about 900 g m^−2^. For each species, three plots were randomly selected. In each plot, three soil cores (4 cm diameter) were collected at 0–20 cm depth after the removal of the above‐ground plant parts. These three cores were mixed to form a single sample. In total, 33 soil samples (11 grassland species × 3 samples) were obtained.

### 2.3 Measurement of Soil Aggregate Stability and Chemical Properties

The wet‐sieving method was employed to determine the distribution of water‐stable soil aggregates (WSA) (Deviren Saygin et al. 2012). Briefly, 50 g of air‐dried soil from each sample was submerged in water for 5 min and then subjected to vertical oscillation (3 cm amplitude) for 10 min using an aggregate stability analyser. Aggregates were fractionated into seven size classes: > 2 mm, 2–1 mm, 1–0.5 mm, 0.5–0.25 mm, 0.25–0.106 mm, 0.106–0.053 mm and < 0.053 mm. Each fraction was oven‐dried at 105°C to constant weight. The soil pH and electrical conductivity (EC) were determined in a 1:5 soil/water suspension (Hasbullah and Marschner 2015). Total nitrogen (TN) and total carbon (TC) were determined through combustion using a carbon and nitrogen analyser (Elementar Vario El Cube, Hanau, Germany) (Thuille et al. 2015). Soil organic carbon (SOC) was determined by dichromate oxidation method (Walkley 1947). The Mo‐Sb antifouling spectrophotometry method was used to determine the total phosphorus (TP). The NaHCO_3_ leaching method was employed for measuring the available phosphorus (AP) (Yang et al. 2011). The available nitrogen (NH+ 4‐N and NO– 3‐N) was determined using a chemical analyser (Smartchem140, AMS, Italy) (Song et al. 2013). The soil potassium content was determined using the Soil Available Potassium Assay Kit (Solarbio, BJ, CN) (Bai et al. 2015).

### 2.4 High‐Throughput Sequencing

Total DNA was extracted from 0.5 g soil samples using the Omega Mag‐Bind DNA Kit for Soil (Omega Bio‐Tek, Norcross, GA, USA) according to the instructions of the manufacturer. The quantity and quality of the extracted DNA were assayed using a Nanodrop ND‐2000 UV–VIS spectrophotometer (NanoDrop Technologies, Wilmington, DE, USA). The V1 region of the fungal ITS (Internally Transcribed Spacer) was amplified with the primers ITS1F: GGAAGTAAAAGTCGTAACAAGG and ITS2R: GCTGCGTTCTTCATCGATGC. The PCR products were then purified using the VAHTS DNA Clean Beads (Vazyme Biotech, NJ, JS, CN) and quantified with the Microplate Reader FL×800 (Bio‐Tek, Winooski, Vermont, US). Sequencing was performed on the Illumina MiSeq platform (Illumina, San Diego, CA, USA) at Shanghai Personal Biotechnology Co. Ltd., Shanghai, China. After sequencing, the data were processed using the DADA2 plugin within the QIIME 2 platform (https://qiime2.org/) (Bolyen et al. 2019). The sequences obtained through DADA2 were referred to as amplicon sequence variants (ASVs). Taxonomic classification of the ASVs was performed using the VSEARCH consensus taxonomy classifier available in QIIME2 (version2019.4). Fungal taxonomies were assigned based on the UNITE database (version 8.0 https://unite.ut.ee/).

### 2.5 Data Analyzes

The value of > 0.25 mm water‐stable aggregate (WSA), mean weight diameter (MWD), and geometric mean diameter (GMD) was calculated using the following equation:

$$\mathrm{WSA}\left(\%\right)=\frac{M_{r>0.25}}{M_{T}}$$

$$\mathrm{MWD}=\frac{\sum_{i=1}^{n}\left({\bar{x}}_{i}\cdot w_{i}\right)}{\sum_{i=1}^{n}w_{i}}$$

$$\mathrm{GMD}=\mathrm{EXP}\left[\frac{\sum_{i=1}^{n}w_{i}\;\ln{\bar{x}}_{i}}{\sum_{i=1}^{n}w_{i}}\right]$$

where *n* is the number of aggregate size fractions, *M*_r_ > 0.25 is the mass of aggregates with particle size > 0.25 mm, *M*_T_ is the total mass of aggregates, *W*_i_ is the proportion of the total aggregates in the *i* ^th^ size fraction, *X*_i_ is the mean diameter of the *i* ^th^ size fraction.

The differences in soil aggregate stability and chemistry properties were performed by analysis of variance (ANOVA) and post hoc Tukey's test (*p* < 0.05) using the R ‘agricolae’ package. All data are presented as mean ± standard deviation of three replicates. Dissimilarity of soil physicochemical properties was determined with analysis of similarities (Anosim) and visualised with Principal Component Analysis (PCA) using the ‘vegan’ package in R.

Before sequencing data analysis, all ASVs were rarefied using a rarefaction method to ensure that they were analysed at the same sequencing depth. Dissimilarity of fungal communities was assessed using non‐metric multidimensional scaling (NMDS) based on Bray‐Curtis distances using the ‘vegan’ package in R. The linear discriminant analysis Effect Size (LEfSe) was performed at all classification levels to explore fungal communities with a linear discriminant analysis threshold of 2 using the ‘ggtree’ package in R. Mantel test based on Pearson's correlation (999 permutations) was used to identify the relationship between soil aggregate stability, chemical properties and fungal communities using the ‘linkET’ package in R. The co‐occurrence networks were constructed with R package ‘picante’, ‘reshape2’ and ‘dplyr’ based on a Spearman correlation matrix. When assessing microbial associations within the network, ASVs found in more than 20% of samples were retained. The *p* value for the network was < 0.05 adjusted for false discovery rate (FDR). The *r* value for the network was 0.6 based on random matrix theory (RMT).

Gephi v. 0.10.1 was used to visualise the fungal network and classify the modules using the community detection algorithm with randomise and edge weights. The resolution for modularity was 1.0. The relative abundance of each module was obtained by adding the relative abundances assigned to each ASV assigned to the module (Li et al. 2024). The topological roles of individual nodes were assessed using within‐module connectivity (Zi) and among‐module connectivity (Pi) calculated in R with the ‘reshape2’ and ‘ggrepel’ packages. Nodes were selected based on the thresholds of Zi = 2.5 and Pi = 0.62. ZIPI was visualised with the R package ‘ggplot’. To further investigate the relationship between soil aggregate stability or chemical properties and fungal modules, FUNGuild was used to predict trophic modes (https://github.com/UMNFuN/FUNGuild). Mantel test (the same method as above) was used to identify the relationship between soil aggregate stability, chemical properties and fungal modules. To analyse the differences in soil aggregate stability, chemical properties and fungal trophic modes, we performed a one‐way ANOVA and conducted a post hoc Tukey's test (*p* < 0.05) using the ‘car’ package in R.

## 3 Results

### 3.1 Effects of Plant Species on Soil Aggregate Stability and Chemical Properties

Planting 11 native grassland species revealed clear interspecific differences in their effects on soil chemical properties (Table 1), including EC, TC, TN, NH+ 4, TP, AP and AK, while collectively leading to distinct changes in overall soil properties compared with abandoned land (Figure S1). The highest soil EC was observed in *A. laxm* grassland. *E. dahu* grassland exhibited markedly superior soil TC. *E. dahu* and *A. frig* grasslands showed the highest soil TN among 11 grassland species. *A. gmel*, *A. frig*, *S. offi* and *P. chin* grasslands showed higher soil NH+ 4 than other grassland species. *E. sibi*, *L. chin*, *E. dahu* and *A. laxm* grasslands showed higher soil TP than other grassland species. *L. chin* and *S. offi* grasslands show higher soil AP than other grassland species. The highest soil AK was observed in *L. chin* grassland.

**TABLE 1:** Soil chemical properties of 11 grassland species.

Species	pH	EC (μs·cm^−1^)	TC (g·kg^−1^)	SOC (g·kg^−1^)	TN (g·kg^−1^)	NO– 3 (mg·kg^−1^)	NH+ 4 (mg·kg^−1^)	TP (g·kg^−1^)	AP (mg·kg^−1^)	AK (mg·kg^−1^)
*L. chin*	5.79 ± 0.04	18.96 ± 0.57**abc**	6.99 ± 0.34**b**	2.70 ± 0.23	0.72 ± 0.06**ab**	7.01 ± 0.74	7.70 ± 0.19**c**	0.35 ± 0.03**ab**	15.71 ± 0.74**a**	35.28 ± 1.11**a**
*A. hirt*	5.70 ± 0.01	17.44 ± 2.01**bc**	5.91 ± 0.35**cde**	2.25 ± 0.30	0.55 ± 0.03**c**	5.22 ± 0.20	7.77 ± 0.23**c**	0.26 ± 0.01 **cd**	8.03 ± 0.33 **fg**	22.23 ± 0.58**bcd**
*E. kamo*	5.73 ± 0.04	16.32 ± 0.68**c**	5.33 ± 0.15**de**	3.73 ± 1.34	0.57 ± 0.02**c**	5.01 ± 0.50	8.42 ± 0.46**bc**	0.24 ± 0.02 **cd**	7.02 ± 0.57**gh**	25.21 ± 3.30**abcd**
*E. sibi*	5.81 ± 0.05	16.31 ± 0.60**c**	6.29 ± 0.53**bcd**	2.53 ± 0.11	0.59 ± 0.03**c**	6.00 ± 0.91	7.75 ± 0.60**c**	0.37 ± 0.00**a**	10.14 ± 0.79**de**	24.14 ± 2.18**bcd**
*E. dahu*	5.46 ± 0.21	15.43 ± 1.42**c**	8.03 ± 0.46**a**	2.87 ± 0.21	0.79 ± 0.06**a**	4.92 ± 1.00	8.41 ± 0.31**bc**	0.35 ± 0.02**ab**	7.08 ± 1.07**gh**	30.88 ± 2.00**ab**
*A. laxm*	5.69 ± 0.05	23.75 ± 3.57**a**	4.97 ± 0.09**e**	2.42 ± 0.37	0.61 ± 0.02**bc**	8.43 ± 0.32	8.42 ± 0.69**bc**	0.33 ± 0.02**ab**	8.76 ± 0.76**efg**	24.85 ± 2.57**abcd**
*L. bico*	5.68 ± 0.01	22.83 ± 1.71**ab**	5.59 ± 0.07**cde**	2.82 ± 0.10	0.57 ± 0.03**c**	7.05 ± 1.71	8.56 ± 1.10**bc**	0.3 ± 0.02**bc**	11.03 ± 0.27 **cd**	28.68 ± 3.94**abc**
*A. gmel*	5.75 ± 0.10	17.95 ± 1.91**bc**	6.62 ± 0.39**bc**	2.82 ± 0.10	0.61 ± 0.02**bc**	6.28 ± 1.20	11.44 ± 1.59**a**	0.26 ± 0.03 **cd**	5.25 ± 0.27 **h**	25.49 ± 2.07**abcd**
*A. frig*	5.65 ± 0.16	17.04 ± 0.48**c**	6.64 ± 0.22**bc**	2.53 ± 0.06	0.74 ± 0.01**a**	5.61 ± 0.86	11.1 ± 0.74**ab**	0.21 ± 0.00**d**	9.47 ± 0.79**def**	16.27 ± 0.61**d**
*S. offi*	5.58 ± 0.03	20.89 ± 1.97**abc**	6.36 ± 0.04**bcd**	2.42 ± 0.15	0.56 ± 0.01**c**	8.19 ± 0.60	9.11 ± 0.66**abc**	0.25 ± 0.02 **cd**	14.06 ± 0.44**ab**	30.03 ± 7.92**abc**
*P. chin*	5.70 ± 0.02	18.25 ± 1.60**abc**	5.84 ± 0.43**cde**	2.42 ± 0.06	0.63 ± 0.06**bc**	8.68 ± 1.60	10.06 ± 1.29**abc**	0.25 ± 0.01 **cd**	12.64 ± 0.74**bc**	19.46 ± 0.55 **cd**

*Note:* Different bold letters are expressed as significantly different (*p* < 0.05).

Abbreviations: *A. frig*: *Artemisia frigida* ; *A. gmel*: *Artemisia gmelinii* ; *A. hirt*: *Arundinella hirta*; *A. laxm*: *Astragalus laxmannii* ; AK: soil available potassium. *L. chin*: *Leymus chinensis*; AP: soil available phosphorus; *E. dahu*: *Elymus dahuricus* ; *E. kamo*: *Elymus kamoji*; *E. sibi*: *Elymus sibiricus* ; EC: soil electrical conductivity; *L. bico*: *Lespedeza bicolor* ; NH+ 4: soil ammonium nitrogen; NO– 3: soil nitrate nitrogen; *P. chin* : *Potentilla chinensis*; *S. offi*: *Sanguisorba officinalis* ; SOC: soil organic carbon; TC: soil total carbon; TN: soil total nitrogen; TP: soil total phosphorus.

WSA, MWD and GMD of *A. laxm* grassland were higher than those of other species, although no significant differences were found. WSA of *L. chin* grassland, MWD and GMD of *A. frig* were lower than those of other species (Table 2).

**TABLE 2:** Soil aggregate stability of 11 grassland species.

Species	WSA	MWD	GMD
*L*. *chin*	0.70 ± 0.05	0.56 ± 0.04	0.46 ± 0.04
*A. hirt*	0.81 ± 0.02	0.66 ± 0.06	0.52 ± 0.04
*E. kamo*	0.76 ± 0.05	0.64 ± 0.15	0.52 ± 0.10
*E. sibi*	0.75 ± 0.04	0.56 ± 0.03	0.49 ± 0.04
*E. dahu*	0.75 ± 0.01	0.59 ± 0.01	0.47 ± 0.01
*A. laxm*	0.87 ± 0.04	0.73 ± 0.17	0.62 ± 0.13
*L. bico*	0.82 ± 0.05	0.57 ± 0.06	0.48 ± 0.05
*A. gmel*	0.75 ± 0.02	0.63 ± 0.08	0.50 ± 0.05
*A. frig*	0.77 ± 0.05	0.50 ± 0.05	0.42 ± 0.03
*S. offi*	0.78 ± 0.06	0.53 ± 0.04	0.45 ± 0.02
*P. chin*	0.78 ± 0.03	0.56 ± 0.07	0.47 ± 0.04

Abbreviations: GMD: geometric mean diameter; MWD: mean weight diameter; WSA: water‐stable aggregate.

**FIGURE 1:**
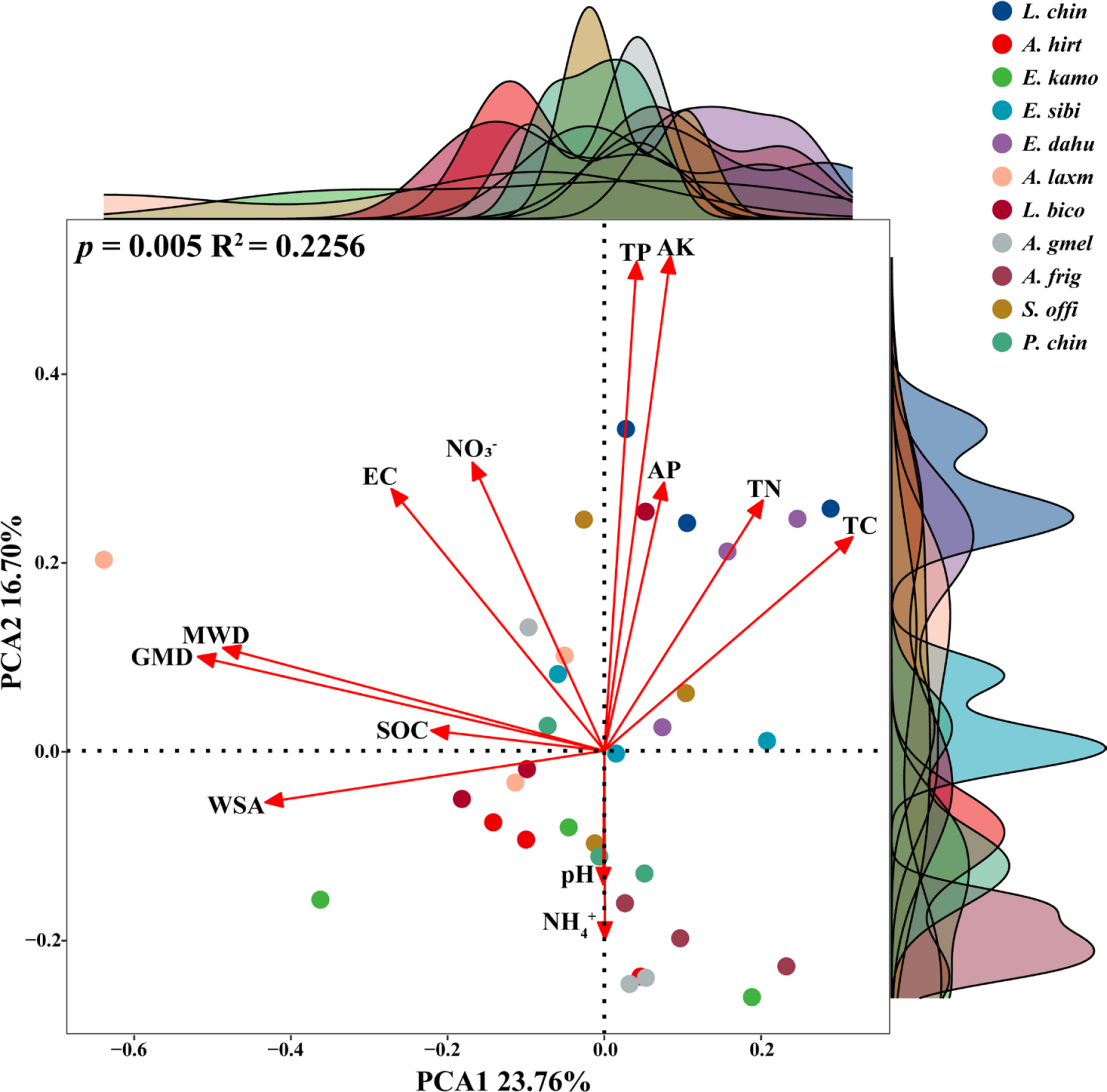
Anosim and PCA of soil aggregate stability and chemical properties. Curves along the PCA axes depict the density distribution of the different plant species.

Principal Component Analysis (PCA) revealed distinct soil property variations across 11 plant species (Figure 1). The first two axes of the PCA analysis explained 40.46% of the total variation (PCA1, 23.76%; PCA2, 16.70%). Soil aggregate stability parameters (WSA, GMD, MWD) loaded predominantly on PCA1, while chemical properties (TP, AK, AP, pH, NH+ 4) were aligned with PCA2. Consistent with this spatial separation, Anosim confirmed significant multivariate differences in both soil aggregate stability and chemical properties among the 11 plant species.

### 3.2 Effects of Plant Species on Soil Fungal Community

**FIGURE 2:**
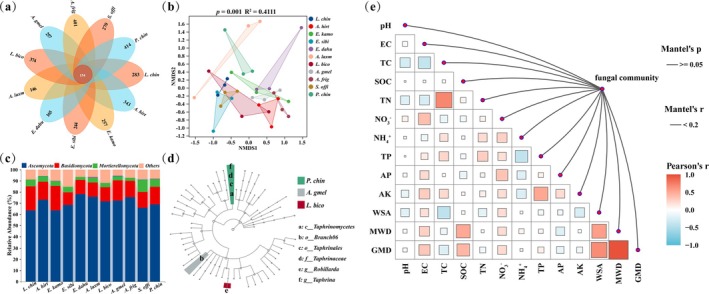
Soil fungal communities of 11 grassland species. (a) Venn plots of soil fungi. (b) Anosim and NMDS of fungal communities based on the Bray‐Curtis distance at the ASV level. (c) The relative abundance of the dominant fungal phyla. (d) LEfSe of soil fungi based on all classification levels. (e) Relationship between soil aggregate stability, chemical properties and soil fungal communities based on Pearson's correlation. Black lines from the fungal community indicate non‐significant correlations (*p* > 0.05).

A total of 4848 fungal sequences were detected obtained across all planting treatments, with 154 common sequences (Figure 2a). The unique sequences of *L. chin*, *A. hirt*, *E. kamo*, *E. sibi*, *E. dahu*, *A. laxm*, *L. bico*, *A. gmel*, *A. frig*, *S. offi* and *P. chin* were 283, 343, 257, 244, 165, 146, 374, 257, 401, 270 and 414, respectively. A significant difference was observed in the composition of fungal communities among 11 plant species (Figure 2b). At the phylum level, fungal communities were dominated by Ascomycota (~70.65%), Basidiomycota (~15.56%), and Mortierellomycota (~4.94%) (Figure 2c), all exhibiting notably higher relative abundances compared to abandoned soil controls (Figure S2). The relative abundance of Mortierellomycota was significantly higher in *S. offi* grassland than in other grasslands (*p* < 0.05). A total of two orders, one class, one family and two genera were identified as biomarkers among the 11 grasslands at all taxonomic levels (Figure 2d). The relative abundance of Taphrinales, Taphrinomycetes, Taphrinaceae and Taphrina were found to be abundant in *P. chin* (*p* < 0.05). The relative abundance of Branch06 exhibited a notable increase in *A. frig* (*p* < 0.05), while Robillarda displayed a significant enrichment in *L. bico* (*p* < 0.05). Changes in soil fungal communities are not influenced by a single soil physicochemical property (Figure 2e).

### 3.3 Associations Between Soil Aggregate Stability, Chemical Properties and Fungal Ecological Modules

**FIGURE 3:**
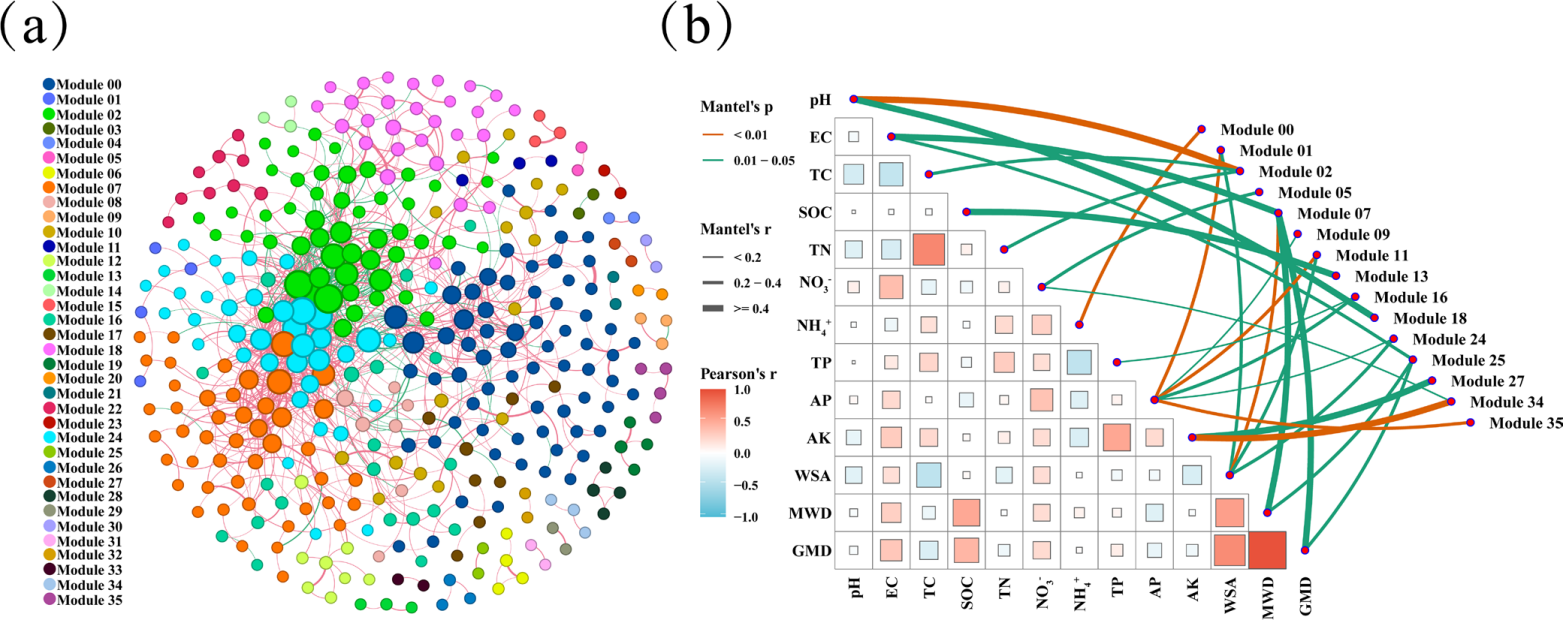
Modular analysis of co‐occurrence network under 11 grassland species on the basis of ASV. (a) Co‐occurrence network and ecological modules of soil fungi. Nodes in each network are coloured based on their respective module. Red edges connecting nodes represent co‐occurrence interactions, and green lines indicate mutual exclusion. (b) Relationship between soil aggregate stability, chemical properties and soil fungal modules based on Pearson's correlation (*p* < 0.05).

A fungal co‐occurrence network comprising 36 modules with 366 nodes and 1078 edges was constructed from all ASV sequences of 11 grasslands (Figure 3a; Table S1). The majority of nodes in the microbial network are classified as peripherals (~95.50%) (Pi < 0.62, Zi < 2.5), connected only to nodes within their own module (Figure S3). Connectors and module hubs account for 3.55% and 0.55% of all ASVs respectively. A total of 13 connectors are distributed within modules 00, 02, 08, 16, 17, 18 and 24, and two module hubs are distributed within modules 07 and 08. According to the Mantel test, soil aggregate stability and chemical properties are all significantly related to fungal module communities (Figure 3b). Among the soil chemical properties, pH is significantly correlated with modules 02 and 18. EC is significantly correlated with modules 07 and 25. TC and TN are significantly correlated with module 02. SOC is significantly correlated with module 13. NH+ 4is significantly correlated with module 00. NO– 3 is significantly correlated with module 05. TP is significantly correlated with module 16. AP is significantly correlated with modules 01, 09, 11, 16, 24 and 35. AK is significantly correlated with modules 27 and 34. Module 07, which is significantly correlated with the soil chemical property EC, and modules 01, 11 and 24, which are significantly correlated with AP, are also regulated by WSA. Similarly, module 07, which is significantly correlated with the soil chemical property EC, and module 25, which is significantly correlated with the soil chemical property AP, are affected by MWD and GMD.

### 3.4 Trophic Modes in Modules Related to Soil Aggregate Stability and Chemical Properties

**FIGURE 4:**
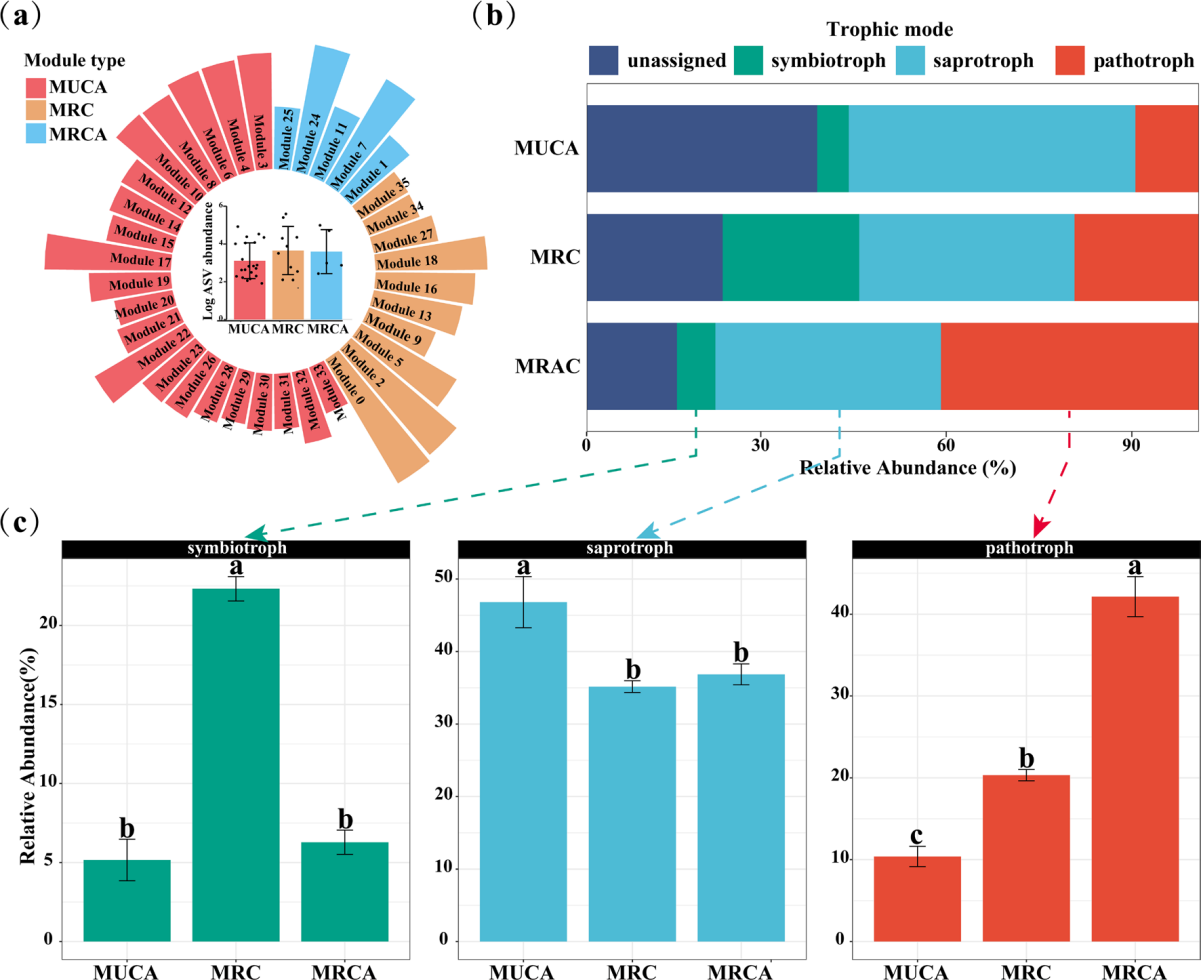
Module characteristics of co‐occurrence network based on the relationship between soil chemical properties or aggregate stability and fungal module. (a) The outer circle shows the relative abundance of each module contained in different module types; the inner circle represents the average relative abundance of different module types; the relative abundance of a single module is equal to the sum of the relative abundances of the ASVs it contains, taken logarithmically. (b) Trophic mode composition of different type modules based on FUNGuild. (c) One‐way ANOVA and Tukey's test (*p* < 0.05) in the same trophic modes among different module types. MRC: modules related to soil chemistry; MRCA: modules related to soil chemistry and aggregate stability; MUCA: modules unrelated to soil chemistry or aggregate stability.

According to the relationships between modules and soil chemical properties or aggregate stability, all modules within the fungal network were classified into three types: Modules Unrelated to Soil Chemical Properties or Aggregate Stability (MUCA), Modules Related to Soil Chemical Properties (MRC) and Modules Related to Soil Chemical Properties and Aggregate Stability (MRCA). The average relative abundances of MUCA, MRC and MRCA are 3.11, 3.65 and 3.60, respectively. (after taking logarithm) (Figure 4a). The fungal trophic modes in MUCA, MRC and MRCA were obtained from FUNGuild, which revealed a difference in the proportion of fungal trophic modes among the modules (Figure 4b). In the MUCA, the relative abundance of pathotroph, saprotroph and symbiotroph is 10.39%, 46.81% and 5.16% respectively. In the MRC, the relative abundance is 20.34% for pathotroph, 35.16% for saprotroph, and 22.32% for symbiotroph. In the MRCA, the relative abundance is 42.14% for pathotroph, 36.85% for saprotroph, and 6.28% for symbiotroph. Furthermore, we conducted a one‐way ANOVA to compare the three trophic modes across different module types (Figure 4c). The relative abundance of saprotroph in the MUCA was found to be significantly higher than that in both the MRC and MRCA. Additionally, the relative abundance of symbiotroph was significantly higher in the MRC compared to the MUCA and MRCA. In the MRCA, the relative abundance of pathotroph was significantly higher than in the MRC, while the abundance of pathotroph in the MRC was significantly higher than in the MUCA.

## 4 Discussion

Planting native grassland species on degraded soil reveals significant species‐specific variation in their capacity to maintain or improve soil service functions. In our study, the 11 grassland species exerted distinct influences on soil physicochemical properties and fungal communities, while soil physicochemical properties did not directly drive corresponding shifts in overall fungal community structure. Notably, the modular organization of microbial co‐occurrence networks effectively uncovered plant‐specific linkages between soil physicochemical properties and fungal community responses. Modules were classified according to their relationships with soil properties as follows: Modules Unrelated to Soil Chemical Properties or Aggregate Stability (MUCA), Modules Related to Soil Chemical Properties (MRC) and Modules Related to Soil Chemical Properties and Aggregate Stability (MRCA). These linkages were further associated with the distribution of fungal trophic modes within modules. For instance, the proportion of symbiotic fungi was significantly higher in MRC compared with other modules, whereas the proportion of pathogenic fungi was significantly higher in MRCA. Our findings highlight that the modular pattern of microbial networks provides a novel perspective for understanding the coupling mechanisms between soil properties and microbial communities under plant species establishment, and offer theoretical support for the restoration and management of abandoned land and other disturbed lands.

### 4.1 Do Plant Species Shape Fungal Communities Through Soil Aggregate Stability and Chemical Properties?

The relationship between plants and soil is intricate and complex (Custódio et al. 2022). It is believed that plants can influence soil chemical properties through their nutrient absorption strategies (Peñuelas et al. 2019), litters (Hobbie 2015) and root exudates (Ma, Tang, et al. 2022). In our research, significant differences in soil chemical properties, including EC, TC, TN, NH+ 4, TP, AP and AK, were observed among the 11 grassland plant species. Also, the fungal communities of the 11 species were significantly different. *S. offi* grassland significantly increased the relative abundance of Mortierellomycota, a dominant phylum in grasslands, and the microorganisms within this phylum may enhance nutrient uptake by forming symbiotic relationships with plants (Wei et al. 2024; Wu et al. 2021). This suggests that the planting of *S. offi* may result in positive feedback that enhances soil restoration and facilitates plant growth. On the contrary, *P. chin* significantly increased the relative abundance of Taphrinales, Taphrinomycetes, Taphrinaceae and Taphrina. Most of the Taphrinales are fungal pathogens, such as Taphrina, which can induce deformities in host plants (Rossi et al. 2007; Tsai et al. 2014). Previous studies also suggest that plants of the genus *Potentilla* are considered host plants for the pathogenic fungus Taphrina (Bacigálová et al. 2014; Petrýdesová et al. 2016). This may be one of the reasons why *P. chin* occurs in the natural grassland communities of the study area predominantly as a companion species and in small sizes. It can also be inferred that *P. chin* is not an effective resource for soil microbial remediation.

From the perspective of microbial community analysis, it is hypothesized that the influence of plants on soil properties can significantly alter the composition of fungal communities (Duchicela et al. 2013; Lange et al. 2019). This is based on the plants' physiological processes (Liu et al. 2019; van der Putten et al. 2016), such as the release of root exudates (McLaughlin et al. 2023), which can change the soil physicochemical properties and specific fungal communities. In our study, although significant differences in soil fungal communities were observed among the 11 species, we did not find any correlation between the entire fungal communities and soil aggregate stability or chemical properties. This may be due to the short timeframe (two growing seasons) being inadequate to establish persistent plant‐induced coupling between soil physicochemical properties and fungal assemblages. To better understand the deep relationship between soil physicochemical properties and fungal communities in short‐term revegetation grasslands, a detailed and integrated approach is required.

### 4.2 Do Fungal Communities Exhibit Modularity With Respect to Soil Aggregate Stability and Chemical Properties?

In natural environments, microorganisms rarely exist in isolation; instead, they frequently form multiple ecological clusters (Bagnaro et al. 2020). Network analysis identifies node clusters as modules based on microbial interaction relationships (Srinivasan et al. 2024). Independent modules of the microbial network tend to be more closely linked to soil multiple ecological functions than the whole microbial community (Zhang, Feng, et al. 2024). In our experiments, all soil aggregate stability parameters or chemical properties were significantly correlated with specific soil fungal modules. Our results support De Menezes's view that modules based on network analysis can more accurately reflect the detailed relationship between soil physicochemical properties and microorganisms (De Menezes et al. 2015). In other words, although we did not observe that plants regulate entire soil fungal communities by soil aggregate stability or chemical properties in the short term, we did confirm that the effects of revegetated grasslands on soil physicochemical properties would affect the soil fungal communities at the modular level. Moreover, we found an interesting phenomenon: soil aggregate stability is not related to the modules alone. Soil aggregates, an organic combination of soil solids and pores, are not readily amenable to alteration in the immediate term (Wang et al. 2018), which may limit its effect on soil fungal communities. This absence of association between fungal communities and soil properties has rarely been mentioned in previous studies.

### 4.3 Do the Trophic Modes in the Fungal Module Explain the Relationship Between Modules and Soil Aggregate Stability or Chemical Properties?

In order to explore the reasons for the individual or combined effects of soil aggregate stability and chemical properties on the fungal module, we divided all modules into MUCA (Modules Unrelated to Soil Chemistry or Aggregate stability), MRC (Modules Related to Soil Chemistry) and MRCA (Modules Related to Soil Chemistry and Aggregate stability). We classified the fungi within each module into pathotroph, symbiotroph, saprotroph according to their trophic modes (Nguyen et al. 2016). The FUNGuild and one‐way ANOVA results show that trophic modes differ significantly among the MUCA, MRC and MRCA. In our research, 21 modules were not associated with the soil aggregate stability or chemistry properties. We speculate that the phenomenon is due to the higher proportion of saprophytic fungi in MUCA. Soil saprophytic fungi, which act as miners in the soil, can decompose organic or inorganic substances and convert them into nutrients that can be used by other organisms (Cao et al. 2024). Their survival adaptability largely depends on plant‐derived organic matter, such as leaf and root litter (Bonanomi et al. 2017; Shahzad et al. 2015). In previous research, Francioli et al. also showed that the traits of plant species explain the saprotrophic fungal community better than the soil physicochemical properties (Francioli et al. 2021).

A total of ten modules were classified as MRC, including module 35, module 34, module 27, module 18, module 16, module 13, module 9, module 5, module 2 and module 0. MRC showed higher relative abundance in soil symbiotic fungi than MUCA and MRCA. Soil symbiotic fungi, such as arbuscular mycorrhizal fungi, are known to establish symbiotic relationships with plants (Wipf et al. 2019). These relationships confer a range of benefits to both parties while being influenced by soil chemical properties (Bennett and Groten 2022; Ma et al. 2023). This result may be related to the regulatory role of soil nutrient availability in the plant‐fungi symbiosis (Zhang, Ndungu, et al. 2024). For example, under conditions of phosphorus limitation, symbionts maintain a relatively high abundance by forming a symbiotic relationship with plants (Ferrol et al. 2019; van't Padje et al. 2021). Soil pH and EC have a substantial impact on the mobility of ions in soil dilutions and their uptake by plants and soil fungi (Jamiołkowska et al. 2018; Lenoir et al. 2016). In forest ecosystems, soil pH is considered an important factor mediating the relationship between tree species and their associated symbiotic fungi (Urbanová et al. 2015). Similarly, high soil electrical conductivity inhibits the growth of symbiotic fungi and alters their community composition (Kewessa et al. 2024; Singleton et al. 1982).

The MRCA contains five network modules: module1, module7, module11, module24 and module25. We can clearly observe a stepwise increase in the relative abundance of pathogenic fungi among different trophic mode modules. The relative abundance of pathogenic fungi in MRCA is significantly higher than that in MRC, while the relative abundance in MRC is significantly higher than that in MUCA. It has been suggested that the enhanced soil nutrients, particularly through the addition of nitrogen and phosphorus, can create an environment that favors the proliferation and dissemination of pathogenic fungi (Lekberg et al. 2021). On the other hand, soil aggregates and the pore structures they form can act as shelters that reduce plant susceptibility to pathogenic fungi (Erktan et al. 2020). These aggregates may also impede the diffusion and transport of the plant's volatile fungicides, thereby suppressing the plant's defense response and creating conditions conducive to the survival of pathogenic fungi (Schmidt et al. 2015). In any case, the sensitivity of pathogens to soil chemistry and aggregate stability highlights the intricate relationship between soil properties and fungal dynamics. We speculate that soil aggregate stability selectively regulates the soil fungal communities by providing specific microhabitats that cater to the needs of certain fungal groups, including pathogenic fungi. Given that pathogenic fungi require suitable growth conditions based on soil chemical properties, and that soil aggregates offer favorable spatial conditions, it is plausible that aggregates play a key role in modulating fungal community composition and pathogen prevalence. It is worth noting that soil pathogens have the potential to impact plant growth and yield by infecting the root system and subsequently spreading to the aboveground parts (Mommer et al. 2018). More importantly, pathogens can enter animals and humans through the food chain and cause a range of diseases, posing a direct and specific threat to plant, animal and human health (Banerjee and van der Heijden 2023). With the improvement of soil structure caused by increasing the size of soil aggregates during the revegetation process, we should closely monitor the change trends of pathogenic fungi in the soil.

## 5 Conclusions

Overall, short‐term grassland species planting exerts a considerable influence on soil aggregate stability, chemical properties and fungal communities. However, no correlation exists between soil aggregate stability and chemical properties, nor do they correlate with the entire soil fungal community. Significantly, both parameters demonstrated established associations with fungal modular assemblages. Soil chemical properties regulated the network module dominated by symbiotic fungi, while soil chemical properties and aggregate stability jointly regulated the network module dominated by pathogenic fungi. The network module dominated by saprophytic fungi is not regulated by soil aggregate stability or chemical properties. More importantly, we found that soil aggregate stability did not affect the soil fungal communities alone in the short term, and they did not show a significant effect even at the module level. These findings offer a new perspective on the relationship between soil aggregate stability, chemical properties and fungal communities in the short‐term revegetation grasslands, and provide a theoretical basis for revegetation and soil management following disturbance.

## Author Contributions

The research was designed by J.L. Samples were collected by Z.D. and J.L. Z.D. performed the laboratory work, conducted the analyses, and wrote the manuscript with assistance from J.L. and L.B. Sequence processing, data curation, and data analyses were carried out by both Z.D. and J.L. J.L. supervised the entire study. All authors approved the final manuscript.

## Conflicts of Interest

The authors declare no conflicts of interest.

## Supporting information


**Figure S1:** Changes in soil physicochemical properties following the planting of native grassland species. The adjacent abandoned land was used as a control. Data are shown as mean response ratios (RR) ± SE. An RR value above zero indicates that the property is higher under grassland species planting compared with the control, whereas an RR value below zero indicates lower values relative to the control. The same applies below.
**Figure S2:** Changes in soil fungal phylum following the planting of native grassland species. The adjacent abandoned land was used as a control. Data are shown as mean RR ± SE.
**Figure S3:** Distribution of fungal ASVs based on their network roles. Nodes in the network are classified as peripherals, module hubs, network hubs or connectors.
**Table S1:** Topological properties of fungal co‐occurrence network under 11 revegetation grasslands.

## Data Availability

ITS gene sequences were deposited to the Sequence Read Archive (SRA) under the project accession number PRJNA1206529.
