# Supplementary material for: Ecological Modules Link Soil Aggregate Stability, Chemical Properties and Fungal Communities Under Plant Species‐Based Revegetation
Source: Environ Microbiol Rep. 2025 Nov 4;17(6):e70228. doi: 10.1111/1758-2229.70228 (PMC12586355; doi:10.1111/1758-2229.70228)
Supplement: Supplementary file 1 — Figure S1: Changes in soil physicochemical properties following the planting of native grassland species. The adjacent abandoned land was used as a control. Data are shown as mean response ratios (RR) ± SE. An RR value above zero indicates that the property is higher under grassland species planting compared with the control, whereas an RR value below zero indicates lower values relative to the control. The same applies below. Figure S2: Changes in soil fungal phylum following the planting of native grassland species. The adjacent abandoned land was used as a control. Data are shown as mean RR ± SE. Figure S3: Distribution of fungal ASVs based on their network roles. Nodes in the network are classified as peripherals, module hubs, network hubs or connectors. Table S1: Topological properties of fungal co‐occurrence network under 11 revegetation grasslands. [file EMI4-17-e70228-s001.docx]

**
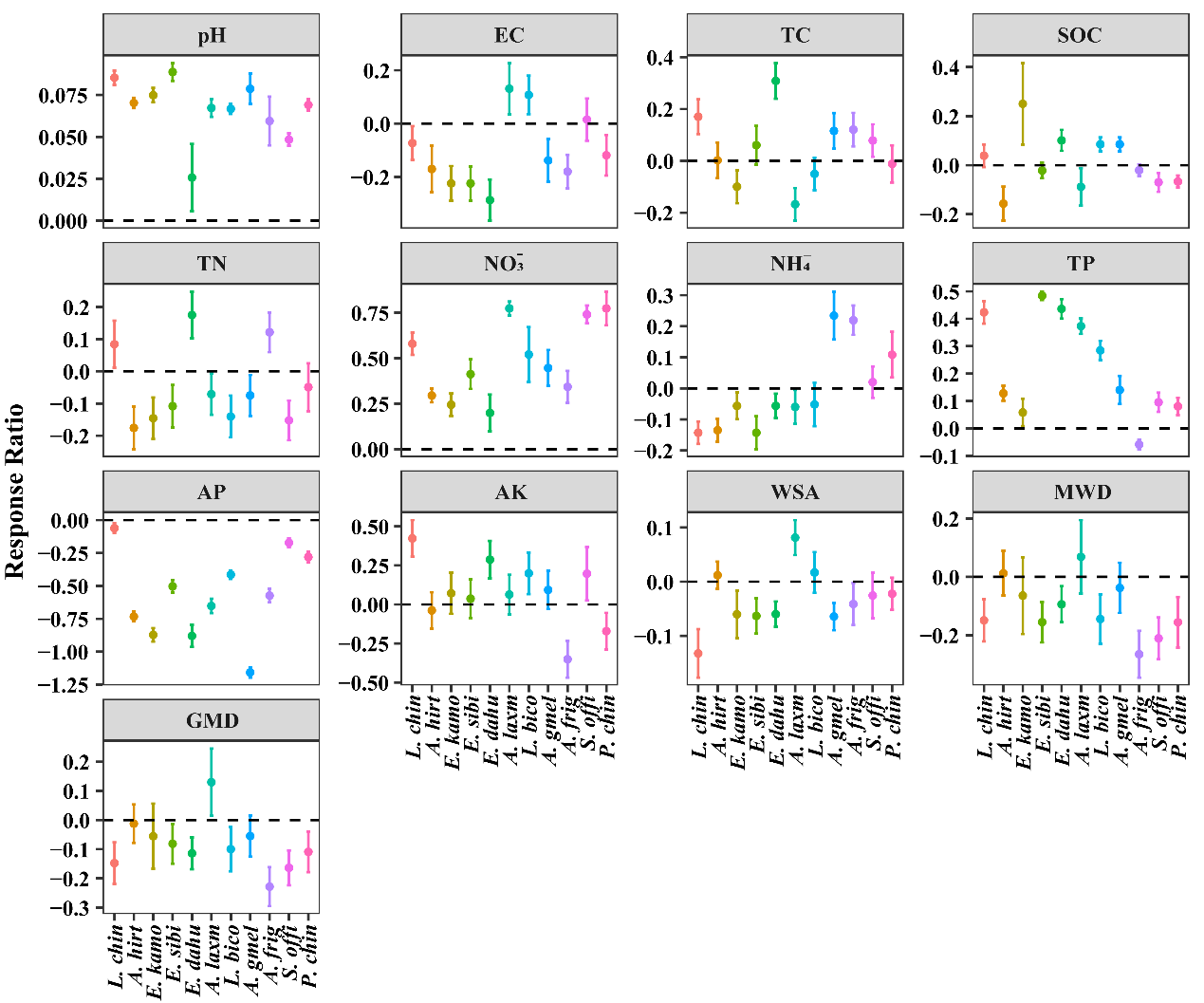
**

**Fig. S1.** Changes in soil physicochemical properties following the planting of native grassland species. The adjacent abandoned land was used as a control. Data are shown as mean response ratios (RR) ± SE. An RR value above zero indicates that the property is higher under grassland species planting compared with the control, whereas an RR value below zero indicates lower values relative to the control. The same applies below.

**
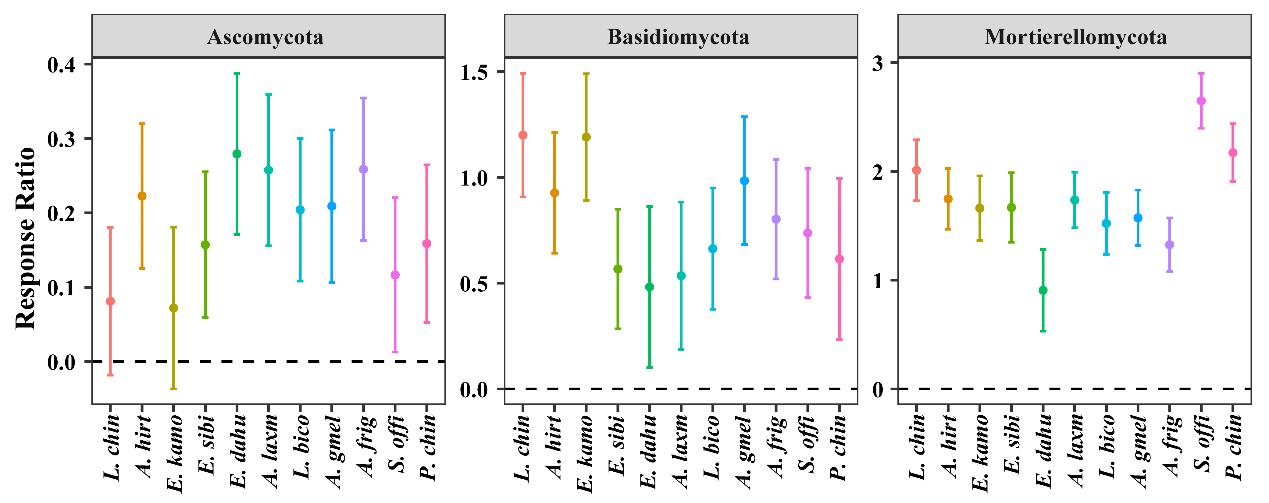
**

**Fig. S2.** Changes in soil fungal phylum following the planting of native grassland species. The adjacent abandoned land was used as a control. Data are shown as mean RR ± SE.

**
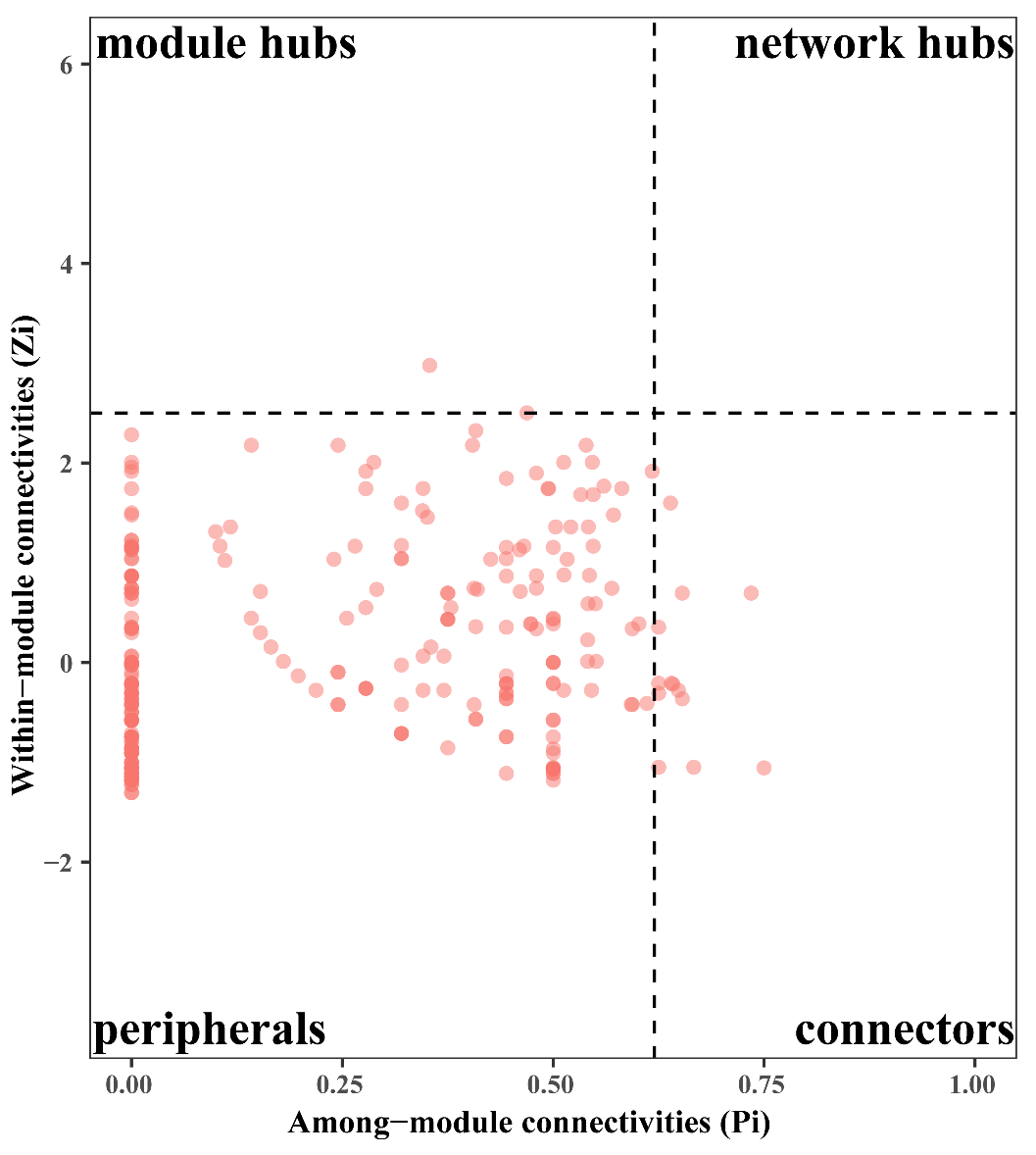
Fig. S3** Distribution of fungal ASVs based on their network roles. Nodes in the network are classified as peripherals, module hubs, network hubs or connectors.

**Table S1** Topological properties of fungal co-occurrence network under eleven revegetation grasslands.

| Nodes | Edges | Ecological clusters | Average degree | Average weighted degree | Network diameter | Graph density | Modularity | Average clustering coefficient | Average path length |
| --- | --- | --- | --- | --- | --- | --- | --- | --- | --- |
| 366 | 1078 | 36 | 5.891 | 3.923 | 12 | 0.016 | 0.533 | 0.323 | 4.319 |
